# Dissecting genetic loci affecting grain morphological traits to improve grain weight via nested association mapping

**DOI:** 10.1007/s00122-019-03410-4

**Published:** 2019-08-09

**Authors:** Xiaoqian Wang, Luhao Dong, Junmei Hu, Yunlong Pang, Liqin Hu, Guilian Xiao, Xin Ma, Xiuying Kong, Jizeng Jia, Hongwei Wang, Lingrang Kong

**Affiliations:** 1 State Key Laboratory of Crop Biology, Shandong Key Laboratory of Crop Biology, College of Agronomy, Shandong Agricultural University, Tai’an, 271018 China; 2 Key Laboratory of Crop Gene Resources and Germplasm Enhancement, Ministry of Agriculture, The National Key Facility for Crop Gene Resources and Genetic Improvement, Institute of Crop Sciences, Chinese Academy of Agricultural Sciences, Beijing, 100081 China

**Keywords:** Wheat, Nested association mapping, Grain morphology, Grain weight, QTL

## Abstract

**Key message:**

The quantitative trait loci (QTLs) for grain morphological traits were identified via nested association mapping and validated in a natural wheat population via haplotype analysis.

**Abstract:**

Grain weight, one of the three most important components of crop yield, is largely determined by grain morphological traits. Dissecting the genetic bases of grain morphology could facilitate the improvement of grain weight and yield production. In this study, four wheat recombinant inbred line populations constructed by crossing the modern variety Yanzhan 1 with three semi-wild wheat varieties (i.e., Chayazheda, Yutiandaomai, and Yunnanxiaomai from Xinjiang, Tibet, and Yunnan, respectively) and one exotic accession Hussar from Great Britain were investigated for grain weight and eight morphological traits in seven environments. Eighty-eight QTLs for all measured traits were totally identified through nested association mapping utilizing 14,643 high-quality polymorphic single nucleotide polymorphism (SNP) markers generated by 90 K SNP array. Among them, 64 (72.7%) QTLs have the most favorable alleles donated by semi-wild wheat varieties. For 14 QTL clusters affecting at least two grain morphological traits, nine QTL clusters were located in similar position with known genes/QTL, and the other five were novel. Three important novel QTLs (i.e., *qTGW-1B.1*, *qTGW-1B.2*, and *qTGW-1A.1*) were further validated in a natural wheat population via haplotype analysis. The favorable haplotypes for these three QTLs might be used in marker-assisted selection for the improvement of wheat yield by modifying morphological traits.

**Electronic supplementary material:**

The online version of this article (10.1007/s00122-019-03410-4) contains supplementary material, which is available to authorized users.

## Introduction

As one of the world’s major staple crops, wheat (*Triticum aestivum* L.) is critical for global food security (Li and Yang 2017). To feed the growing human population with decreasing area of land, wheat yield must be improved substantially (Simmonds et al. 2016). Grain weight, an important component of grain yield, is mainly determined by grain morphological traits, such as grain length, grain width, and grain area (Gegas et al. 2010). Therefore, dissecting the genetic basis of grain morphological traits is crucial for the improvement of grain weight and yield.

Grain morphology and weight are traits of quantitative inheritance, and many QTLs affecting wheat grain morphological traits and grain weight have been identified (Cheng et al. 2017; Cui et al. 2014; Heidari et al. 2011; Huang et al. 2003, 2006, 2004; Liu et al. 2014; Mccartney et al. 2005; Rasheed et al. 2014; Remington et al. 2001; Ren et al. 2018; Risch and Merikangas 1996; Stich et al. 2010; Wang et al. 2011; Wu et al. 2015; Yan et al. 2017; Yu et al. 2008; Zanke et al. 2015). Two complimentary tools that have been used to dissect the genetic architecture of complex quantitative traits are linkage mapping and genome-wide association studies (Risch and Merikangas 1996). Linkage mapping, possessing high power for QTL identification (Stich et al. 2010), has been extensively applied in the detection of QTL for wheat grain morphology and weight using biparental populations, such as recombinant inbred line (RIL) (Cheng et al. 2017; Jia et al. 2013; Liu et al. 2014; Wu et al. 2015), *F*_2_ (Yan et al. 2017), *F*_2:3_ (Wang et al. 2011), doubled haploid (DH) population (Heidari et al. 2011; Huang et al. 2006; Mccartney et al. 2005), and advanced backcross populations (Huang et al. 2003, 2004). Compared with linkage mapping, a genome-wide association study has a higher resolution for QTL detection (Remington et al. 2001). Recently, association studies have been applied in the analysis of grain morphology and weight in wheat (Rasheed et al. 2014; Zanke et al. 2015). To combine the advantages of two mapping methods, a nested association mapping (NAM) strategy was proposed for the genetic dissection of complex traits using several RIL populations derived from multiple-cross mating with a shared parent (Yu et al. 2008). However, only a few studies on QTL mapping through the NAM strategy in wheat have been conducted (Cui et al. 2014; Ren et al. 2018).

The increase in crop yield potential mainly benefited from artificial domestication (breeding). Grain morphology and weight have undergone a positive selection via open environments, which is one of the main components of domestication syndrome (Brown et al. 2009; Fuller 2007). Compared with ancestral wheat species, domesticated wheat species have a larger grain size (Fuller 2007). However, the crop domestication results in the improvement of grain yield but at a cost of reduced genetic variation which is referred to as the “domestication bottleneck” (Dempewolf et al. 2017). Luckily, genetic diversity is preserved in the wild relatives of wheat. Thus, exploiting and utilizing wild resources could break through the bottleneck in wheat breeding. Over the past few decades, the utilization of wild species in wheat breeding has achieved success. For instance, Tang et al. (2014) isolated a line from the cross between common wheat and *Thinopyrum intermedium* (Host) Barkworth and Dewey. This line carried the translocation of the *T. intermedium* chromosome replacing chromosome 6A of common wheat, which is highly resistant to powdery mildew. Similarly, a wheat *Leymus mollis* (Trin.) Pilg. 3D (3Ns) substitution line replacing chromosome 3D of common wheat was isolated by Pang et al. (2014). It was characterized by resistance to leaf rust and improved spike length and spike number. Zhang et al. (2014) developed some translocation lines carrying seed storage protein genes from chromosome 1 V of *Dasypyrum villosum*, which showed positive effects on qualities in wheat that are exploited for bread-making.

Yunnan wheat (*Triticum aestivum* ssp. *yunnanense* King), Tibetan wheat (*Triticum aestivum* ssp. *tibetanum* Shao), and Xinjiang wheat (*T. petropavloski* Udatsz. et Migusch.) are three unique wheat subspecies that were discovered in western China (Wang et al. 2007). Yunnan wheat, characterized by tough and hard glumes, is a semi-wild cultivar that was discovered in Yunnan (Dong et al. 1981). It has the advantages of good quality, preharvest sprouting resistance, high tolerance to poor soil fertility, drought, cold, and frost and is considered one of the most precious resources for wheat breeding (Chen et al. 2007; Dong et al. 1981). Tibetan wheat is primitive semi-wild hexaploid wheat from Tibet (Shao et al. 1980), which has a spike morphology similar to that of common wheat but is resistant to abiotic stresses and has a strong seed dormancy and strong nutrition deficiency tolerance (Liu et al. 2014; Sun et al. 1998). As one of the Chinese endemic wheat landraces, Xinjiang wheat, known as “Daosuimai,” or rice-head wheat, is characterized by a long glume (Chen et al. 2013). It is a hexaploid wheat species that was discovered in the Talimu basin of Xinjiang, and it has a similar morphology as the tetraploid *T. turgidum* ssp. *polonicum*. These semi-wild wheat varieties possess many advanced traits; however, only a few studies have been conducted on mining the favorable alleles of semi-wild wheat cultivars to control grain morphology and weight (Liu et al. 2014; Luo et al. 2016).

Therefore, to better exploit desirable QTL/genes from wild relatives and exotic germplasms for grain morphology and weight, four RIL populations were constructed, with a modern wheat variety Yanzhan 1 (YZ) as the common female parent and four male parents including (three unique semi-wild subspecies from China and one dwarf cultivar Hussar from Great Britain). Nested association mapping was performed to identify the favorable alleles for wheat grain weight and grain morphological traits. Some important QTLs were further validated in a wheat natural population. Our study could help to provide a better understanding of the genetic base that controls grain morphological traits; therefore, it would be helpful for the genetic improvement of wheat grain weight and yield potential.

## Materials and methods

### Plant materials

The study comprised four RIL populations derived from crosses between YZ (the common female parent) and four semi-wild or exotic germplasms. YZ, a high-yielding and good-quality winter wheat variety released in Henan province of the Huanghuai region in China, possessed small seed size. The four male parents included Hussar (HR, a dwarf winter wheat cultivar) from Great Britain and three semi-wild wheat varieties including Yunnanxiaomai (YN, *T. aestivum* ssp. *yunnanense* King, semi-winterness variety) from Yunnan, Chayazheda (CY, *T. aestivum* ssp. *tibetanum* Shao, winter variety) from Tibet, and Yutiandaomai (YT, *T. aestivum*. *petropavloski* Udats et Migusch, winter variety) from Xinjiang. Single seed descent method was applied to develop the RIL populations by eight times of self-pollination from F_2_ generation. The population sizes for YN, CY, YT, and HR populations were 97, 82, 96, and 94, respectively.

### Field trials

The four RIL populations, along with their corresponding parents, were planted in seven environments in the Shandong Province of China in two locations Heze (115.51°E, 35.58°N) and Dezhou (116.39°E, 37.38°N) in 2015 and 2016 and one location Taian (117.17°E, 36.17°N) from 2015 to 2017. The environment names were coded by year of evaluation and abbreviation of location name, e.g., 15H refers to environment in 2015 at Heze. Each line was planted in a two-row plot with 50 seeds per row with a row length of 2.0 m and a row spacing of 0.25 m. Two replicates were performed under each environment. The local farmers’ standard management practices were followed for the field of each environment.

### Trait measurements

At maturity, 10 uniform plants in the middle of each plot were bulk harvested. The seeds were fully cleaned and dried before trait measurement. The thousand grain weight (TGW, in g), grain length (GL, in mm), grain width (GW, in mm), grain area (GA, in mm^2^), grain perimeter (GP, in mm), grain diameter (GD, in mm), grain shape (GS), and grain roundness (GR) were measured using a grain seed measurement machine (SC-E, Wanshen Technology Company, Hangzhou, China). Due to some uncontrollable factors, the traits for the CY-RIL population in 15 T, 15H, and 15D were not collected.

### Genotyping

The genomic DNA of the four interconnected RIL populations and five parents was extracted from fresh seedling leaves using the CTAB method (Doyle 1987). The qualified DNA was genotyped using the wheat 90 K iSelect array (Wang et al. 2014). The physical positions for all the SNP markers were determined through a BLAST alignment to the IWGSC Reference Sequence v1.0 (Alaux et al. 2016) using the short sequences that harbored SNP loci, and only the uniquely matched SNP loci were retained. The heterozygous alleles were regarded as missing, and the SNP loci with a missing rate over 20% and minor allele frequency (MAF) less than 0.05 were removed. Finally, a total of 14,643 high-quality SNP markers were used in the following analysis.

### Population structure and kinship

The population structure and kinship of the four interconnected RIL populations sharing one common parent (nested association population) were analyzed using the 14,643 high-quality SNP markers. A model-based Bayesian clustering analysis method implemented in STRUCTURE software version 2.3.4 (Pritchard et al. 2000) was utilized for structure analysis. The parameters were set as follows: *k*, the number of groups in the panel varying from 1 to 5; 5 runs each k value; 10,000 burn-in iterations followed by 10,000 MCMC (Markov chain Monte Carlo) iterations. For kinship calculation, the Centered_IBS method implemented in TASSEL5.2.23 was used (Bradbury et al. 2007). The kinship heatmap was constructed using the R package “d3heatmap.”

### Phenotypic analysis

The best linear unbiased prediction (BLUP) across the seven evaluated environments was calculated using the “lmer” function implemented in R package lme4, with both genotypes and environments considered as random factors. Phenotypic correlations were computed using the BLUP value of each line via the “rcorr” function implemented in the R package Hmisc (Harrell and Dupont 2013).

### Nested association mapping

NAM was performed both on the BLUP for each trait from the combined analysis of seven environments and on the mean values for each trait in each individual environment from individual environment analyses using the NAM package (Xavier et al. 2015) in R. In the “NAM” package, an association analysis was carried out through the efficient massive mapping algorithm (EMMA). The P3D strategy was utilized to avoid updating the polygenic term for every marker. Using the empirical Bayes approach, each molecular marker was treated as a random effect, and the model was refitted using Eigen decomposition and evaluated with the likelihood ratio test (Xavier et al. 2015). A QTL was claimed when the test statistics reached *P* < 5 × 10^−3^. The QTLs affecting different traits with overlapped chromosome interval were designated as QTL clusters. In this study, two kinds of QTL clusters were defined. The clusters affecting both grain weight (TGW) and size (GL, GW, GA, GD, or GP), were given the prefix “*QW*,” and the clusters affecting grain shape (GS or GR) and size (GL, GW, GA, GD, or GP) were given the prefix “*QS.*” The annotated genes inside the QTL intervals were extracted from the IWGSC RefSeq Annotations database v1.0 (https://wheat-urgi.versailles.inra.fr/Seq-Repository/Annotations).

### Validation of important QTLs by haplotype analysis

A validation was carried out for important QTLs through haplotype analysis using wheat natural population comprising 574 wheat cultivars or advanced breeding lines originated from 43 regions worldwide. QTLs that met the following criteria were regarded as important: (1) affecting at least four grain-size-related traits simultaneously; (2) being consistently identified in at least three individual environments. First, several KASP (Kompetitive Allele Specific PCR) markers inside the interval of important QTLs were developed from SNP markers linked to the target QTL or SNP markers with the unique position on wheat reference genome selected from Wheat 660 K SNP array designed by the Chinese Academy of Agricultural Sciences and synthesized by Affymetrix. (https://wheat.pw.usda.gov/ggpages/topics/Wheat660_SNP_array_developed_by_CAAS.pdf). Then, all the available KASP markers inside the QTL region were used to genotype 574 wheat cultivars or lines. Finally, haplotype analysis was performed by comparing the significant differences among major haplotypes (containing more than 8 samples) for each important QTL through analysis of variance (ANOVA).

## Results

### Traits variations and correlations

ANOVA results showed that differences among genotypes and environments were highly significant for all measured traits (Table S1). Genotype (G) explained an average of 51.8 ± 6.9% of the phenotypic variance, ranging from 39.4% for GW to 62.6% for GL. Environment (E) accounted for an average of 12.9 ± 5.1% of the phenotypic variation, ranging from 6.0% for GL to 21.8% for GW. G × E interaction was also significant for all measured traits and the phenotypic variance accounted for was 27.9% ± 2.0%, ranging from 25.3% for GP to 30.8% for GR.

**Fig. 1:**
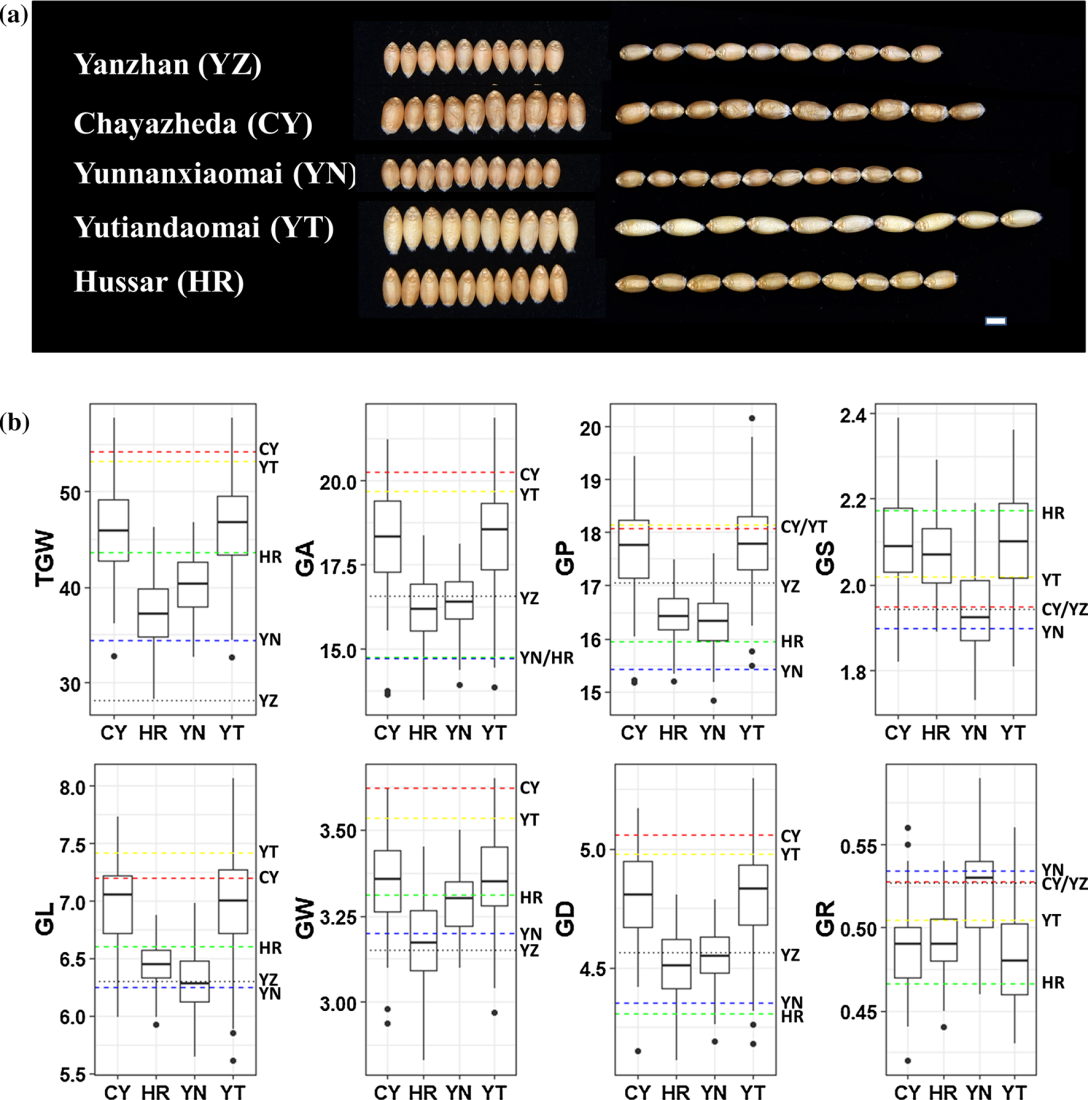
**a** Grain morphology of five parents in Taian of 2017. The scale bar represents 3 mm. **b** Boxplots for eight wheat grain morphology traits and grain weight. CY, HR, YN, and YT represented the RIL populations coming from the cross of the common parent YZ and the corresponding male parent CY, HR, YN, and YT, respectively. TGW, thousand grain weight; GA, grain area; GP, grain perimeter; GS, grain shape; GL, grain length; GW, grain width; GD, grain diameter; GR, grain roundness. The black dotted line represented the common female parent YZ. The dashed line of red, green, blue, and yellow represented the male parent CY, HR, YN, and YT, respectively

**Fig. 2:**
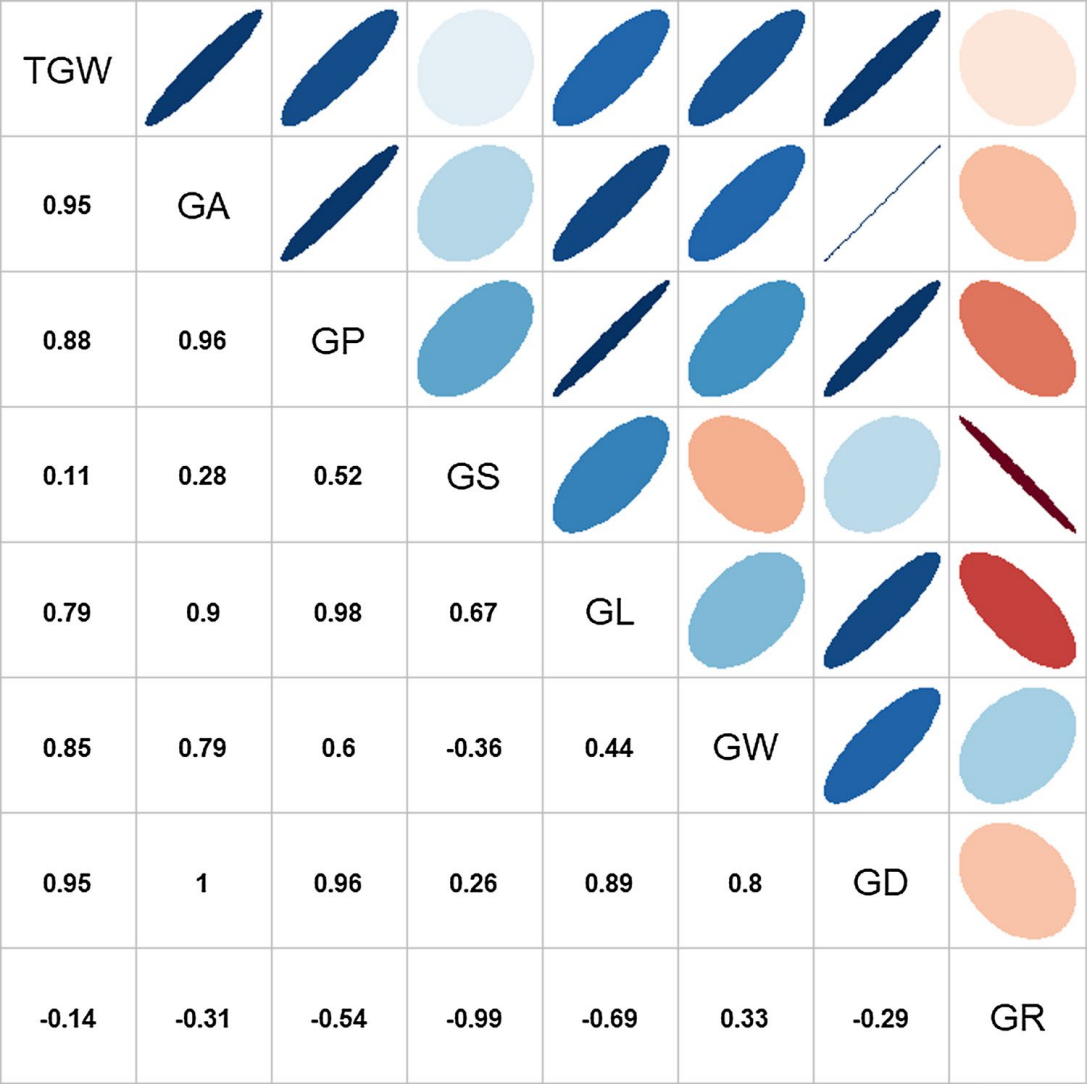
Phenotypic correlations between eight evaluated traits using the BLUP value for each line. In the lower triangular, the values were correlation coefficients (*r*) multiplied by 100. In the upper triangular, the areas and colors of ellipses showed the absolute value of corresponding *r*. Right and left oblique ellipses indicated positive and negative correlations, respectively. The values without glyphs indicated insignificant at 0.01

The BLUP values for each line were used to draw the boxplots and conduct the phenotypic correlation analysis. The five parents showed great differences in grain morphology (Fig. 1). The common parent YZ exhibited a moderate grain size compared with the parent CY, which was characterized by the largest GW, GD, GA, and GP, and the parent YT had the largest GL compared with the other three parents. Thus, the TGW of CY (52.70 g) and YT (49.05 g) was higher, followed by YZ (38.24 g), YN (31.85 g), and HR (29.58 g) (Fig. 1). Wide variations were observed for all measured traits in the four RIL populations. Moreover, most of the traits in each RIL population appeared to be normally distributed, and strong transgressive segregations toward both directions were observed. The trend of differences in mean values among the populations was consistent with the differences among their male parents (Fig. 1). The phenotype pairwise correlations between the eight measured traits are illustrated in Fig. 2. Significant correlations between the traits were observed at the level of *p* = 0.01. The GA, GP, GL, GW, GS, and GD were positively correlated with one another (except for GS with GW), and the correlation coefficients varied from 0.26 of GS with GD to 1.00 of GA with GD. The GR was negatively correlated with all other grain morphological traits except for GW, with correlation coefficients ranging from − 0.29 with GD to − 0.99 with GS. TGW was positively correlated with all the grain morphological traits (except for GR), especially with GA and GD, with correlation coefficients being 0.95 in both cases. To investigate the environmental stability of the measured traits, the correlations between seven environments for each trait were calculated (Fig. S1). For all the traits, the positive correlations between different environments were significant at the level of *p* = 0.01, with most of the correlation coefficients being larger than 0.50. The GL showed the best environmental stability, with correlation coefficients ranging from 0.70 to 0.92, while the GW showed unstable correlation coefficients ranging from 0.25 to 0.79.

### Basic statistics of markers

**Fig. 3:**
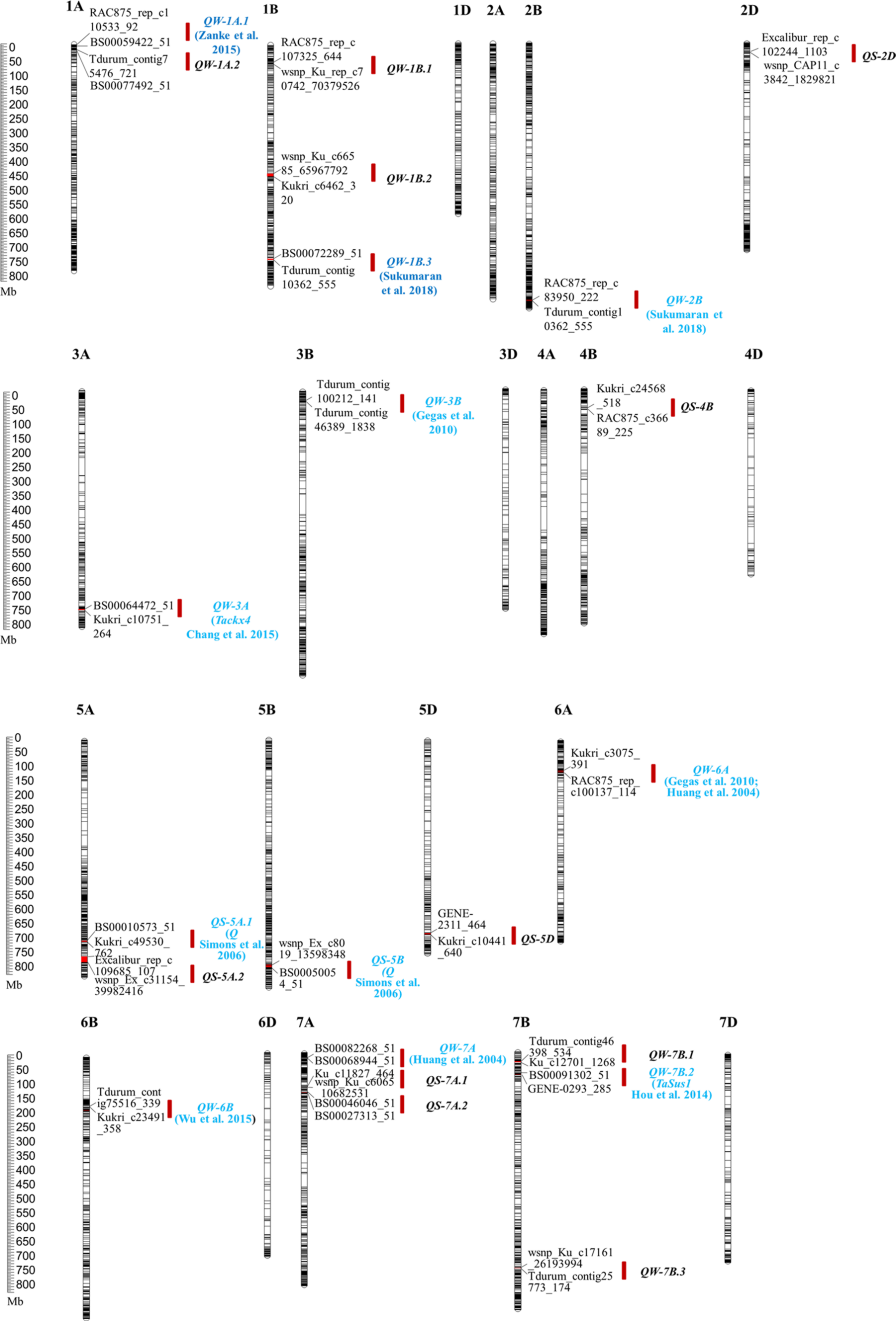
QTL clusters for eight traits evaluated. The QTLs in green were mapped to a similar location with related cloned genes or mapped QTL reported previously

For the 14,643 high-quality SNP markers, there were 5881, 6483, and 2279 markers on sub-genomes A, B, and D, respectively. The number of markers per chromosome ranged from 112 on chromosome 4D to 1292 on chromosome 2B (Table S2 and Fig. 3). The coverage rate of the 14,643 SNP markers was 82.70% on average, ranging from 66.28% for chromosome 6D to 90.48% for chromosome 3A. The average marker spacing was 1.28 Mb, with spacing ranging from 0.60 Mb for chromosome 1A and 4.54 Mb for chromosome 4D. On average, the marker spacing for sub-genomes A, B, and D was 0.87 Mb, 0.85 Mb, and 2.14 Mb, respectively (Table S2).

### Population genetic structure

The entire nested association population comprised four RIL populations. According to values of LnP(D) generated from STRUCTURE, with its modal value used to detect the true k of three groups, *k* = 3 was recommended where the ascent changed gradually according to the method of Evanno et al. (2005). A kinship analysis also suggested three distinct subgroups (Fig. S2). Group I consisted of 75 lines from the CY-RIL population and 90 lines from the YT-RIL population. Group II consisted of 116 accessions, with most of the lines being from the HR-RIL population. Group III comprised 88 lines, and all of them were from the YN-RIL population. In the entire nested association population, 80.2% (296/369) of the lines did not show any admixture, and 6.0% (22/369) showed less than 20% admixture (Fig. S2B).

### QTL mapping

A total of 88 QTLs were identified for eight grain morphological traits through combined analysis of seven environments, which were located on all 21 wheat chromosomes except for 2A, 3D, 4A, 4D, and 7D (Table S3). These QTLs were also detected via individual environment analysis in between one to five environments (Tables 1 and S4). Among the 88 QTLs, 64 (72.7%) had the most favorable alleles (i.e., the largest absolute additive effects in increasing grain weight or decreasing grain shape) donated from the semi-wild cultivars (Tables S3 and S5). CY, YN, and YT contributed the most favorable alleles for 43, 0, and 21 QTLs, respectively. Moreover, the exotic germplasm HR donated the most favorable alleles, amounting to 19 QTLs (21.6%). For the remaining five QTLs (5.7%), the most favorable alleles were from the common parent YZ.

**Table 1:** QTL clusters identified for grain weight or shape in wheat by combined analysis on a nested association population comprised of four RIL populations evaluated under seven environments

QTL clusters	Chr	Interval (bp)	QTL	*P* value	Add_CY^a^	Add_HR^a^	Add_YN^a^	Add_YT^a^	Favorable alleles^b^	No.^c^	QTL/genes reported
*QW-1A.1*	1A	1,339,530–3,556,253	*qGA-1A.1*	0.0001	0.5550	− 0.1290	0.0000	0.2010	CY	3	Zanke et al. (2015)
			*qGD-1A.1*	0.0001	0.0803	− 0.0180	0.0000	0.0290	CY		Heidari et al. (2011)
			*qGP-1A.1*	0.0010	0.2480	− 0.0439	0.0000	0.1130	CY		
			*qGW-1A.1*	0.0005	0.0603	− 0.0196	0.0000	0.0148	CY		
			*qTGW-1A.1*	0.0001	2.2359	− 0.9202	0.0000	0.8276	CY		
*QW-1A.2*	1A	13,980,147–14,155,814	*qGA-1A.2*	0.0030	0.5380	0.0007	− 0.1140	0.2810	CY	3	
			*qGD-1A.2*	0.0013	0.0786	0.0020	− 0.0134	0.0403	CY		
			*qGW-1A.2*	0.0003	0.0635	− 0.0061	0.0151	0.0316	CY		
			*qTGW-1A.2*	0.0011	2.3066	− 0.4433	− 0.1892	1.1295	CY		
*QW-1B.1*	1B	49,926,288–53,251,268	*qGA-1B.1*	0.0000	0.5590	− 0.4200	− 0.4430	0.6910	YT	4	
			*qGD-1B.1*	0.0000	0.0772	− 0.0594	− 0.0630	0.0992	YT		
			*qGL-1B*	0.0015	0.1073	− 0.1054	− 0.0873	0.1563	YT		
			*qGP-1B.1*	0.0001	0.2890	− 0.2520	− 0.2330	0.3920	YT		
			*qTGW-1B.1*	0.0000	1.8450	− 1.4163	− 1.6006	2.7573	YT		
*QW-1B.2*	1B	368,543,950–376,616,816	*qGA-1B.2*	0.0022	0.3810	− 0.7020	0.0000	0.6760	YT	3	
			*qGD-1B.2*	0.0016	0.0532	− 0.1010	0.0000	0.0967	YT		
			*qGP-1B.2*	0.0036	0.2070	− 0.3740	0.0000	0.3720	YT		
			*qTGW-1B.2*	0.0009	1.5048	− 2.8203	0.0000	2.6687	YT		
*QW-1B.3*	1B	612,081,501–613,729,634	*qGA-1B.3*	0.0007	− 0.9050	0.6240	0.0000	0.0000	HR	2	Sukumaran et al. (2018)
			*qGD-1B.3*	0.0008	− 0.1240	0.0873	0.0000	0.0000	HR		
			*qGP-1B.3*	0.0014	− 0.4490	0.2650	0.0000	0.0000	HR		
			*qTGW-1B.3*	0.0004	− 3.7176	2.8905	0.0000	0.0000	HR		
*QW-2B*	2B	775,053,135–775,169,574	*qGA-2B*	0.0005	0.5240	− 0.2750	− 0.1410	0.2910	CY	2	Sukumaran et al. (2018)
			*qGD-2B*	0.0004	0.0727	− 0.0382	− 0.0177	0.0398	CY		
			*qGL-2B*	0.0047	0.0823	− 0.0409	− 0.0359	0.0835	CY		
			*qGP-2B*	0.0008	0.2500	− 0.1390	− 0.0964	0.2050	CY		
			*qTGW-2B*	0.0020	1.6823	− 0.7646	− 0.2833	0.7267	CY		
*QW-3A.1*	3A	688,621,551–688,884,007	*qGA-3A*	0.0037	0.0744	− 0.1150	− 0.0361	− 0.1650	CY	1	*Tackx4* (Chang et al. 2015); Jia et al. (2013); Liu et al. (2014)
			*qGD-3A*	0.0032	0.0099	− 0.0165	− 0.0045	− 0.0228	CY		
			*qTGW-3A*	0.0059	0.4259	− 0.5332	− 0.0911	− 0.6147	CY		
*QW-3B*	3B	27,995,283–29,355,817	*qGD-3B*	0.0049	0.0879	− 0.0350	− 0.0161	0.0292	CY		Gegas et al. (2010)
			*qGW-3B*	0.0008	0.0692	− 0.0268	0.0072	0.0216	CY		
			*qTGW-3B*	0.0020	2.3787	− 0.7884	− 0.2933	0.8805	CY		
*QW-6A*	6A	88,064,319–100,258,094	*qGW-6A.2*	0.0003	− 0.0239	0.0125	0.0022	− 0.0192	HR	4	Gegas et al. (2010); Huang et al. (2004)
			*qTGW-6A*	0.0025	− 0.4970	0.5326	− 0.0830	− 0.7820	HR		
*QW-6B*	6B	146,144,732–147,582,325	*qGW-6B*	0.0002	− 0.0238	0.0138	0.0000	− 0.0195	HR	3	Wu et al. (2015)
			*qTGW-6B*	0.0038	− 0.5134	0.5105	0.0000	− 0.7610	HR		Luo et al. (2016)
*QW-7A*	7A	15,689,345–17,336,520	*qGA-7A*	0.0042	0.6420	− 0.3900	− 0.3820	0.4090	CY		Huang et al. (2004)
			*qGD-7A*	0.0027	0.0931	− 0.0575	− 0.0532	0.0557	CY		Cui et al. (2014)
			*qTGW-7A*	0.0030	2.5507	− 1.7976	− 1.3260	1.3961	CY		
*QW-7B.1*	7B	22,551,193–28,314,004	*qGA-7B.1*	0.0012	0.1840	− 0.0348	0.0000	0.2260	YT	3	
			*qGD-7B.1*	0.0008	0.0265	− 0.0050	0.0000	0.0316	YT		
			*qGP-7B.1*	0.0043	0.0680	− 0.0162	0.0000	0.1360	YT		
			*qTGW-7B.1*	0.0032	0.6905	− 0.1642	0.0000	0.7474	YT		
*QW-7B.2*	7B	64,726,380–64,764,841	*qGW-7B*	0.0042	0.0587	− 0.0215	0.0000	0.0052	CY	2	*TaSus1* (Hou et al. 2014)
			*qTGW-7B.2*	0.0024	1.9665	− 0.7888	0.0000	0.4738	CY		
*QW-7B.3*	7B	627,299,495–629,424,594	*qGA-7B.2*	0.0019	− 0.0784	− 0.0214	− 0.0414	− 0.1800	YZ	1	
			*qGD-7B.2*	0.0027	− 0.0103	− 0.0043	− 0.0047	− 0.0233	YZ		
			*qGP-7B.3*	0.0024	− 0.0393	0.0044	− 0.0310	− 0.1130	YZ		
			*qTGW-7B.3*	0.0043	− 0.3743	− 0.1813	− 0.0790	− 0.4327	YZ		
*QS-2D*	2D	32,472,805–34,231,554	*qGR-2D*	0.0025	− 0.0003	0.0048	0.0011	− 0.0019	HR	3	
			*qGS-2D*	0.0031	0.0008	− 0.0180	− 0.0042	0.0058	HR		
*QS-4B*	4B	23,658,290–25,834,926	*qGR-4B*	0.0026	0.0027	− 0.0058	0.0038	0.0038	YT	3	
			*qGS-4B*	0.0020	− 0.0118	0.0226	− 0.0133	− 0.0162	YT		
*QS-5A.1*	5A	598,078,683–602,784,872	*qGW-5A*	0.0010	− 0.0019	− 0.0156	0.0000	− 0.0024	HR	3	*Q* (Simons et al. 2006)
			*qGS-5A.1*	0.0008	0.0216	− 0.0069	0.0000	0.0059	HR		
*QS-5A.2*	5A	640,940,316–663,284,518	*qGR-5A*	0.0009	− 0.0054	0.0018	0.0000	− 0.0014	HR	2	
			*qGS-5A.2*	0.0009	0.1501	− 0.0517	0.3382	0.7129	HR		
*QS-5B*	5B	643,674,146–658,369,998	*qGR-5B*	0.0009	0.0057	− 0.0013	− 0.0025	0.0029	CY	4	*Q* (Simons et al. 2006)
			*qGS-5B*	0.0005	− 0.0248	0.0052	0.0116	− 0.0126	CY		
*QS-5D*	5D	508,615,719–513,974,882	*qGR-5D*	0.0010	0.0047	− 0.0013	0.0007	0.0012	CY	3	
			*qGS-5D*	0.0005	− 0.0199	0.0049	− 0.0029	− 0.0049	CY		
*QS-7A.1*	7A	108,816,571–109,843,475	*qGR-7A.1*	0.0026	0.0009	− 0.0046	0.0004	0.0065	YT	4	
			*qGS-7A.1*	0.0020	− 0.0027	0.0181	0.0025	− 0.0304	YT		
*QS-7A.2*	7A	126,874,316–126,907,829	*qGR-7A.2*	0.0047	− 0.0017	− 0.0002	0.0034	0.0040	YT	1	
			*qGS-7A.2*	0.0033	0.0091	0.0011	− 0.0137	− 0.0186	YT		

^a^Add_CY, Add_HR, Add_YN, and Add_YT indicated the additive effect in CY-RIL, HR-RIL, YN-RIL, and YT-RIL populations, respectively. Positive values indicate that the YZ alleles increase the corresponding trait, and, conversely, negative values indicate that YZ alleles decrease it; all weights were shown in grams, and all lengths were shown in centimeters

^b^The largest absolute additive effects in increasing grain weight or decreasing grain size

^c^The number of environments in which the corresponding QTL was significantly detected by individual environment analysis

For TGW, 14 QTLs were detected on chromosomes 1A, 1B, 2B, 3A, 3B, 6A, 6B, 7A, and 7B. For seven QTLs, including *qTGW-1A.1*, *qTGW-1A.2*, *qTGW-2B*, *qTGW-3A*, *qTGW-3B*, *qTGW-7A*, and *qTGW-7B.2*, the most favorable alleles were donated by the CY. The favorable alleles of *qTGW-1B.1*, *qTGW-1B.2*, and *qTGW-7B.1* were contributed by YT. For *qTGW-1B.3*, *qTGW-6A*, and *qTGW-6B*, the most favorable alleles were contributed by HR. The most favorable allele of *qTGW-7B.3* was from the common parent YZ.

For GA, 13 QTLs were detected on chromosomes 1A, 1B, 2B, 3A, 5A, 7A, and 7B. The most favorable alleles of seven QTLs (*qGA-1A.1*, *qGA-1A.2*, *qGA-1A.3*, *qGA-2B*, *qGA-3A*, *qGA-5A*, and *qGA-7A*), three QTLs (*qGA-1B.2*, *qGA-1B.1*, and *qGA-7B.1*), two QTLs (*qGA-1A.4* and *qGA-1B.3*), and one QTL (*qGA-7B.2*) were contributed by CY, YT, HR, and YZ, respectively.

For GP, 14 QTLs were identified on chromosomes 1A, 1B, 1D, 2B, 5A, 7A, and 7B. The most favorable alleles of seven QTLs (*qGP-1A.1*, *qGP-1A.2*, *qGP-2B*, *qGP-5A*, *qGP-7A.1*, *qGP-7A.2*, and *qGP-7B.2*), four QTLs (*qGP-1B.1*, *qGP-1B.2*, *qGP-1D*, and *qGP-7B.1*), two QTLs (*qGP-1A.3* and *qGP-1B.3*), and one QTL (*qGP-7B.3*) were donated by CY, YT, HR, and YZ, respectively.

Eight QTLs for GS were identified on chromosomes 2D, 4B, 5A, 5B, 5D, and 7A. The alleles of CY, YT, and HR decreased the GS for two QTLs (*qGS-5B* and *qGS-5D*), three QTLs (*qGS-4B*, *qGS-7A.1, *and *QS-7A.2*), and three QTLs (*qGS-2D*, *qGS-5A.1,* and *qGS-5A.2*), respectively.

Nine QTLs for GL were identified on chromosomes 1A, 1B, 1D, 2B, 5A, 7A, and 7B. The most favorable alleles of six QTLs (*qGL-1A.1*, *qGL-2B*, *qGL-5A*, *qGL-7A.1*, *qGL-7A.2*, and *qGL-7B*), two QTLs (*qGL-1B* and *qGL-1D*) and one QTL (*qGL-1A.2*) were contributed by CY, YT, and HR, respectively.

Nine QTLs for GW were detected on chromosomes 1A, 3B, 5A, 6A, 6B, 6D, and 7B. The most favorable alleles of four QTLs (*qGW-1A.1*, *qGW-1A.2*, *qGW-3B*, and *qGW-7B*), four QTLs (*qGW-6A.1*, *qGW-6A.2*, *qGW-6B*, and *qGW-6D*), and one QTL (*qGW-5A*) were donated by CY, HR, and YZ, respectively.

In total, 14 QTLs for GD were detected on chromosomes 1A, 1B, 2B, 3A, 3B, 5A, 7A, and 7B. The most favorable alleles of eight QTLs (*qGD-1A.1*, *qGD-1A.2*, *qGD-1A.3*, *qGD-2B*, *qGD-3A*, *qGD-3B*, *qGD-5A*, and *qGD-7A*), three QTLs (*qGD-1B.1*, *qGD-1B.2*, and *qGD-7B.1*) and two QTLs (*qGD-1A.4* and *qGD-1B.3*), and one QTL (*qGD-7B.2*) were contributed by CY, YT, HR, and YZ, respectively.

For GR, seven QTLs were detected on chromosomes 2D, 4B, 5A, 5B, and 7A. The alleles of CY, HR, and YT increased the GR for two QTLs (*qGR-5B* and *qGR-5D*), two QTLs (*qGR-2D* and *qGR-5A*) and three QTLs (*qGR-4B*, *qGR-7A.1,* and *qGR-7A.2*), respectively.

### QTL clusters

The 14 QTLs governing TGW were always co-located with the QTL for grain size traits, such as GA, GP, GD, GW, and GL, which merged into 14 QTLs clusters located on chromosomes 1A, 1B, 2B, 3A, 5A, 6A, 6B, 7A, and 7B (Table 1). The semi-wild relatives, CY and YT, contributed the most favorable alleles of the seven and three QTL clusters, respectively, and the exotic line HR donated the most favorable alleles of the three clusters. The other one was from the common parent YZ. *QW-1A.1* consisted of five QTLs for GA, GD, GP, GW, and TGW, and the most favorable alleles were from CY. The cluster *QW-1A.2* for GA, GD, GW, and TGW were detected, and the most favorable alleles were contributed by CY. Four QTL clusters, *QW-1B.3*, *QW-1B.2*, *QW-7B.1*, and *QW-7B.3*, were identified to affect GA, GD, GP, and TGW, and the most favorable alleles were donated by HR, YT, YT, and YZ, respectively. *QW-1B.1* and *QW-2B*, which consisted of QTL for GA, GD, GP, GL, and TGW, were detected, and the most favorable alleles came from YT and CY, respectively. The QTL cluster *QW-3B*, which harbored QTL for GD, GW, and TGW, was identified, with the most favorable alleles contributed by CY. Three QTL clusters for GW and TGW, including *QW-6A*, *QW-6B*, and *QW-7B.2*, were identified, and the most favorable alleles were from HR, HR, and CY, respectively. Two QTL clusters for GA, GD, and TGW, *QW-7A* and *QW-3A.1*, were detected, and the most favorable alleles were contributed by CY.

The QTLs affecting the GS were co-located with QTL for GR or GW, which merged into eight QTL clusters on chromosome 2D, 4B, 5A, 5B, 5D, and 7A (Table 1). The alleles of HR decreased GS for *QS-2D*, *QS-5A.1,* and *QS-5A.2*. For *QS-5B* and *QS-5D*, the alleles decreasing GS were contributed by CY. The alleles of YT decreased GS for *QS-4B*, *QS-7A.1* and *QS-7A.2*.

### Validation and haplotype analysis for important QTLs

**Fig. 4:**
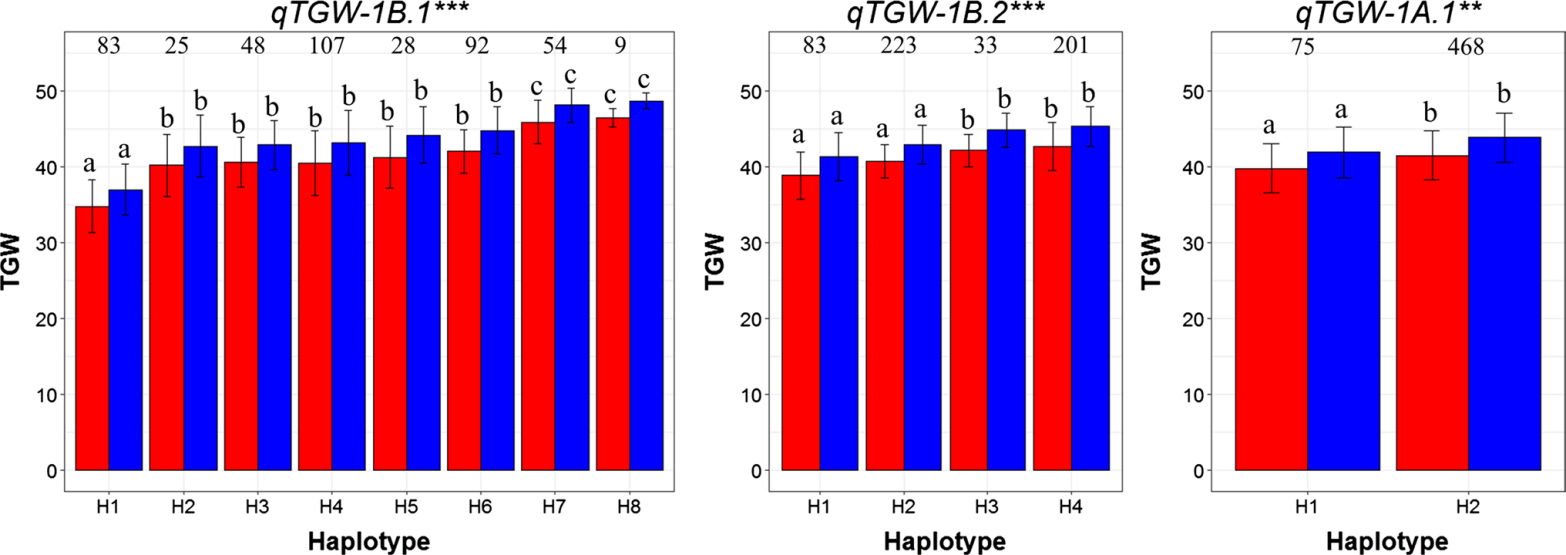
Haplotype analysis for three important QTL *qTGW-1B.1*, *qTGW-1B.2,* and *qTGW-1A.1* using a natural wheat population containing 574 cultivars or lines. The ** and *** suggested significance of ANOVA at *p* < 0.01 and *p* < 0.001, respectively. The letter on histogram (a, b, and c) indicated multiple comparisons result at the significant level 0.01. The red and blue bars indicated the TGW in Taian of 2017 and 2018, respectively

Haplotype analysis was conducted using a natural wheat population containing 574 cultivars or lines to validate three important QTLs: *qTGW-1B.1*, *qTGW-1B.2,* and *qTGW-1A.1* (Table S7). *qTGW-1B.1*, which affected GA, GD, GP, GL, and TGW, was identified in the region of 49,926,288–53,251,268 ( ~ 3.3 Mb) on chromosome 1B in individual environments harboring 30 annotated genes (Tables 1 and S6). This QTL was consistently detected in four individual environments and showed excellent environment stability (Table S4). Subsequently, seven KASP markers in the target region of *qTGW-1B.1* were developed for haplotype analysis through a natural wheat population (Tables S7 and S8). Significant differences were detected for the eight haplotypes at a significant level of *p* = 0.001 (Fig. 4). Haplotypes H7 and H8 showed significantly higher TGW than the other haplotypes. *qTGW-1B.2*, affecting GA, GD, GP, and TGW, was detected in the region of 368,543,950–376,616,816 ( ~ 8.07 Mb) on chromosome 1B in three individual environments harboring 38 annotated genes (Tables 1 and S6). Four KASP markers in the target region of *qTGW-1B.2* were developed for haplotype analysis, and significant differences were detected for the four haplotypes at a significant level of *p* = 0.001 (Tables S7 and S8, Fig. 4). The TGW for haplotypes H3 and H4 was significantly higher than that of haplotypes H1 and H2. For, *qTGW-1A.1*, affecting GA, GD, GP, GW, and TGW, one KASP marker was developed in the target region of 1,339,530–3,556,253 bp ( ~ 2.2 Mb) on chromosome 1A harboring 39 annotated genes (Tables 1 and S6). Haplotype analysis revealed that the two haplotypes showed significant differences at a significant level of *p* = 0.01 (Tables S7 and S8, Fig. 4). Haplotype H2 had a significantly higher TGW than Haplotype H1 did.

## Discussion

### Trait correlations

Grain weight is largely determined by grain morphology. We observed that TGW was highly correlated with the measured grain morphological traits, including GA (0.95), GD (0.95), GP (0.88), GW (0.85), and GL (0.79), which could be partially explained by the co-located QTLs that were identified (Table 1, Fig. 2). The 14 detected QTLs affecting TGW were always co-located with the QTL for the above five morphological traits with the same direction of additive effects. The results suggested that modifying grain morphology is a promising way to increase grain weight. However, the correlations of TGW with GS were very weak, and no QTL affecting both TGW and GS was detected, suggesting that grain weight was not affected by grain shape. Moreover, TGW was more correlated with GW (*r* = 0.85) than with GL (*r* = 0.79), and five and two QTL clusters affecting GW/TGW and GL/TGW were detected, respectively, indicating that GW had stronger effects on TGW than GL did at the QTL level.

The trait correlation results were consistent with previous reports by Cui et al. (2014) and Cheng et al. (2017). However, it was surprising that the correlation between GL and GW was positive (*r* = 0.44), but no QTL affecting both of them was detected; this suggested that the genetic mechanisms of GL and GW may be different and independent. For modern wheat varieties, the accomplishment of high yields benefited from the trade-off among the three yield component traits (spike number per unit, grain number per spike, and grain weight). Thus, the positive phenotypical correlation between GL and GW may have been due to the results of co-directional artificial selection for increasing grain size.

### Comparison with previous studies

In total, 88 QTLs affecting all measured traits were identified, including 14 QTL clusters affecting grain size and weight. Of these, nine clusters were mapped to a similar location, with related cloned genes or mapped QTL reported previously. At the region of QTL cluster *QW-1A.1* detected in the present study, Heidari et al. (2011) detected the QTL region for grain number (GN), fertile spikes/m^2^, and grain weight per spike (GWP) and Zanke et al. (2015) identified the QTL for TGW. The QTL cluster *QW-3A.1*, associated with GA, GD, and TGW in our study, harbored the Cytokinin Oxidase gene *Tackx4*, which has been found to be associated with flag leaf chlorophyll content and TGW (Chang et al. 2015). Moreover, Liu et al. (2014) detected QTL for grain weight per spike and TGW and Jia et al. (2013) identified QTL for grain weight in the same region. In the region of *QW-7B.2*, affecting GW and TGW, wheat sucrose synthase gene *TaSus1* was found; it participated in the conversion of sucrose to starch and was associated with TGW (Hou et al. 2014).

The QTL cluster *QW-1B.3* and *QW-2B*, associated with GA, GD, GP, GL, and TGW, corresponded to the mapped QTL for TGW on chromosome 1B and 2B, respectively, as detected by Sukumaran et al. (2018). *QW-3B* for GD, GW, and TGW corresponded to the QTL region affecting GA, GW, and TGW detected by Gegas et al. (2010). Moreover, *QW-6A* for GW and TGW in the present study were located in the same chromosome region for GW, TGW and factor form density detected by Gegas et al. (2010) and for grain GN, GW, and TGW identified by Huang et al. (2004). At the region of *QW-6B* for GW and TGW in our study, Wu et al. (2015) and Mccartney et al. (2005) identified QTL for GL, GW, GT, and TGW, and Luo et al. (2016) detected QTL for tiller number. *QW-7A*, governing GA, GD, and TGW, was in a similar position as QTL for TGW detected by Cui et al. (2014) and Huang et al. (2004). The other five QTL clusters were located on chromosome regions where no QTL for measured traits has been reported; these can be regarded as novel. The results indicated that a joint multiple related population analysis by NAM was an effective way to detect QTL for interested traits.

Among the eight QTL clusters for grain shape, *QS-5A*, comprising *qGR-5A.2* and *qGS-5A.3*, harbored the major wheat domestication gene *Q*, a member of the APETALA2-like transcription factor family (Simons et al. 2006). The transformation from allele *q* to *Q* on chromosome 5A was found to simultaneously increase GR and decrease GS (Xie et al. 2018). Another two QTL clusters, *QS-5B* and *QS-5D*, covered the homologous genes of *Q* (Simons et al. 2006). Zhang et al. (2011) reported that the chromosome 5A alleles played a key role in domestication-related traits, whereas the chromosome 5B alleles indirectly suppressed the speltoid phenotype, and chromosome 5D alleles were sub-functionalized. The contrasting functions of the chromosome 5A and chromosome 5B alleles for the *Q* gene could explain why the additive effects for *QS-5A.1* and *QS-5B* were opposite in each population. In this study, the alleles from the semi-wild cultivars CY and YT increased the GS and decreased the GR in *QS-5A.1*, which indicated that the domestication from semi-wild cultivar to domesticated cultivar accompanied the transition from slender grains to rounder grains (Charmet 2011; Gegas et al. 2010). Our results support the notion that the domestication of grain size accompanied the formation of free-threshing wheat (Kerber and Rowland 1974; Yan et al. 2017).

### Application potential of semi-wild cultivars in wheat high-yield breeding

Utilizing semi-wild relatives or importing exotic accessions can be an effective way to broaden the genetic diversity of local breeding materials for breeders (Liu et al. 2014; Luo et al. 2016). In this study, we constructed four interconnected RIL populations by crossing local high-yielding wheat variety, YZ, with three semi-wild relatives and one exotic elite variety. These RILs, carrying exotic chromosome fragments, can serve as precious materials for further breeding and genetic studies.

Through NAM for grain morphological traits and grain weight, we could not only identify QTL but also estimate allelic effects from different parents, which gave us the ability to choose the most favorable alleles for wheat high-yield breeding. For the 14 QTL clusters affecting weight detected in the present study, CY, YT, and HR contributed the most favorable alleles for seven, three, and three QTL clusters, respectively, which indicated that these parents carried precious genes for increasing grain weight. Therefore, we could pyramid the most favorable alleles from different parents for high-yield breeding after converting the peak and flanking SNP of identified QTL into other high-throughput genetic markers, such as KASP, to carry out marker-assisted breeding. In this study, three important QTL clusters (*qTGW-1B.1*, *qTGW-1B.2*, and *qTGW-1A.1*) were validated through a natural wheat population containing 574 cultivars or lines via haplotype analysis. The haplotypes of H7/H8, H3/H4, and H2 for *qTGW-1B.1*, *qTGW-1B.2*, and *qTGW-1A.1* showed larger TGW than the other haplotypes did. The KASP markers developed for these three important QTLs could be used for marker-assisted high-yield breeding through the improvement of TGW.

#### Author contribution statement

LK and HW designed the experiments and revise the manuscript. XW analyzed the data and wrote the manuscript. LD, JH, and XM participated in field experiments and the data collection. XW and LD contributed to the genotyping of a natural wheat population. YP assisted in the data analysis and paper revision. XK and JJ provided the RIL populations.

## Electronic supplementary material

Below is the link to the electronic supplementary material.

Supplementary file1 (XLSX 158 kb)

Fig. S1 Phenotypic correlations between seven environments for eight traits. Blue shades indicate significant at P < 0.01. TGW, thousand grain weight; GA, Grain area; GP, Grain perimeter; GS, Grain shape; GL, Grain length; GW, Grain width; GD, Grain diameter; GR, Grain roundness.(TIFF 1399 kb)

Fig. S2 (a) Values of LnP(D) generated from STRUCTURE, with its modal value used to detect the true k of three groups (k = 3). (b) Heat map of kinship from TASSEL with the tree shown on the top and left. (c) Bayesian clustering for NAM populations using STRUCTURE program. (TIFF 526 kb)

Fig. S3 Genotyping results of 12 KASP markers developed for QTL validation by wheat natural populations. (TIFF 3297 kb)
