# Supplementary figures and images for: Dissecting genetic loci affecting grain morphological traits to improve grain weight via nested association mapping
Source: Theor Appl Genet. 2019 Aug 9;132(11):3115–28. doi: 10.1007/s00122-019-03410-4 (PMC6791957; doi:10.1007/s00122-019-03410-4)

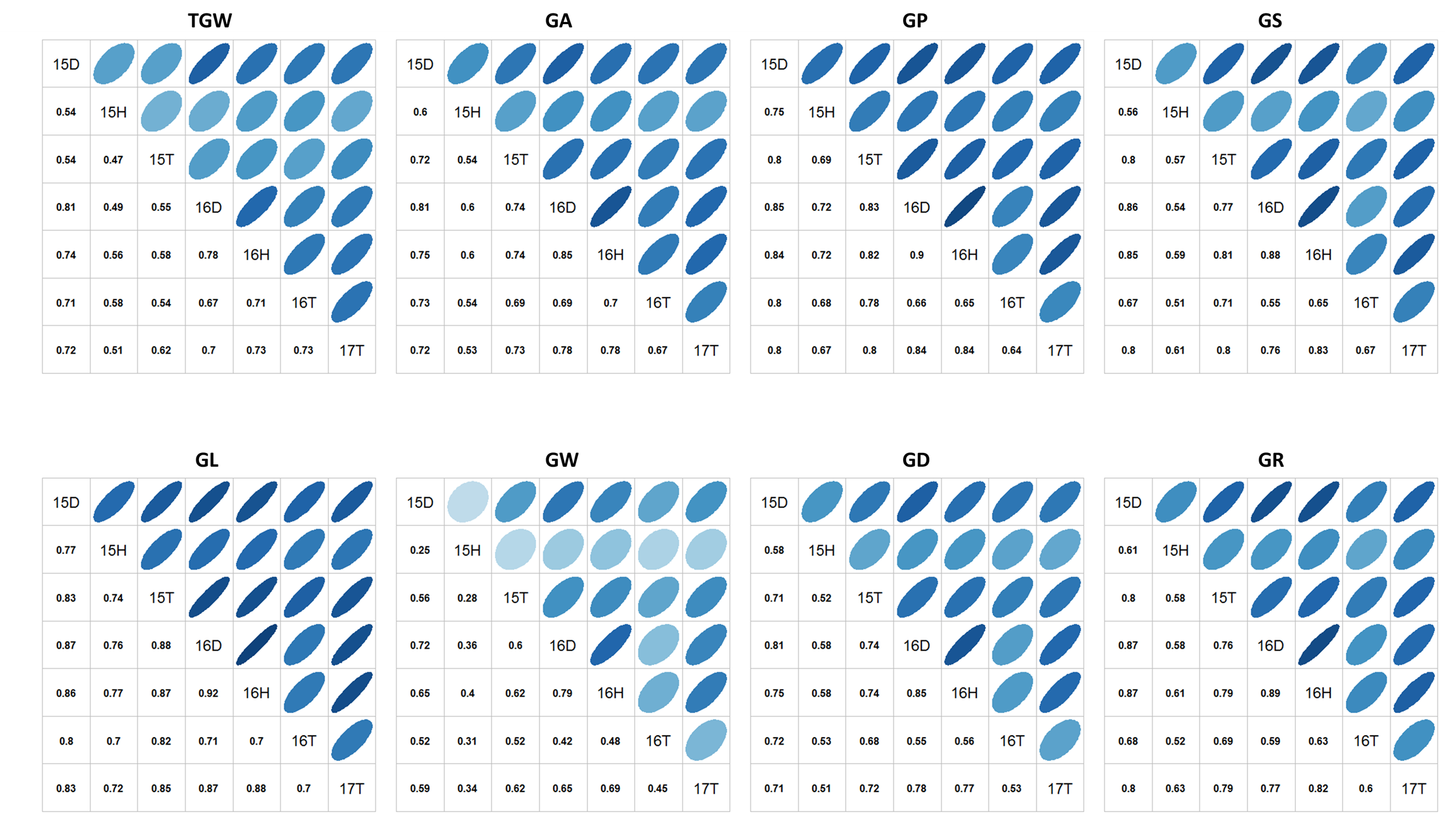

Supplement: Supplementary file 2 — Fig. S1 Phenotypic correlations between seven environments for eight traits. Blue shades indicate significant at P < 0.01. TGW, thousand grain weight; GA, Grain area; GP, Grain perimeter; GS, Grain shape; GL, Grain length; GW, Grain width; GD, Grain diameter; GR, Grain roundness.(TIFF 1399 kb) [file 122_2019_3410_MOESM2_ESM.tif]

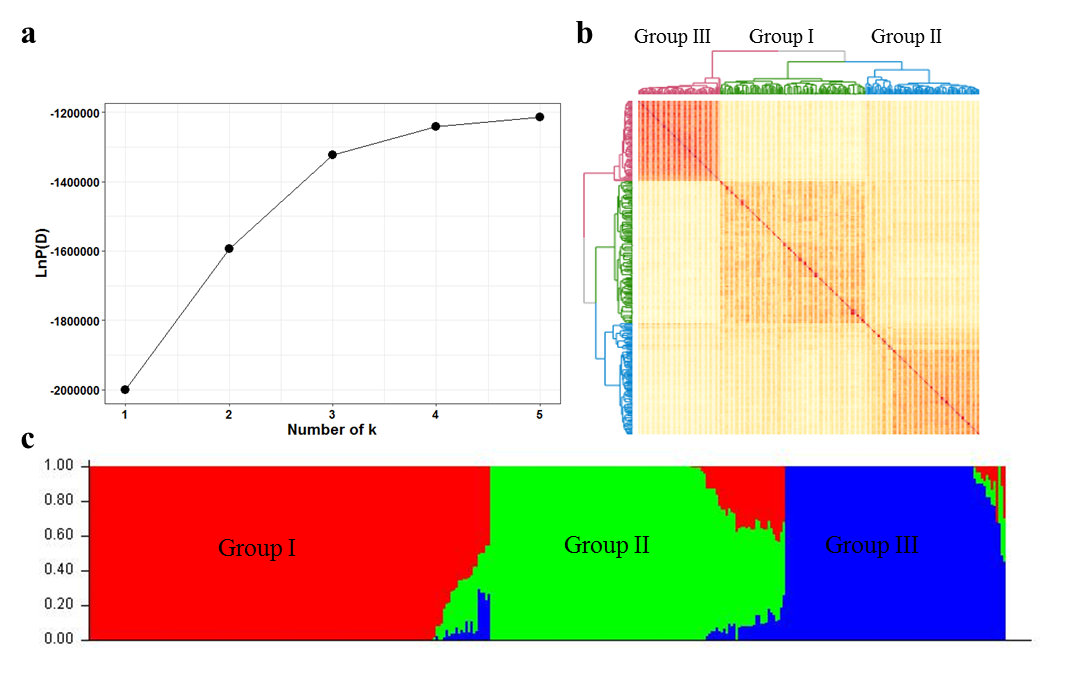

Supplement: Supplementary file 3 — Fig. S2 (a) Values of LnP(D) generated from STRUCTURE, with its modal value used to detect the true k of three groups (k = 3). (b) Heat map of kinship from TASSEL with the tree shown on the top and left. (c) Bayesian clustering for NAM populations using STRUCTURE program. (TIFF 526 kb) [file 122_2019_3410_MOESM3_ESM.tif]

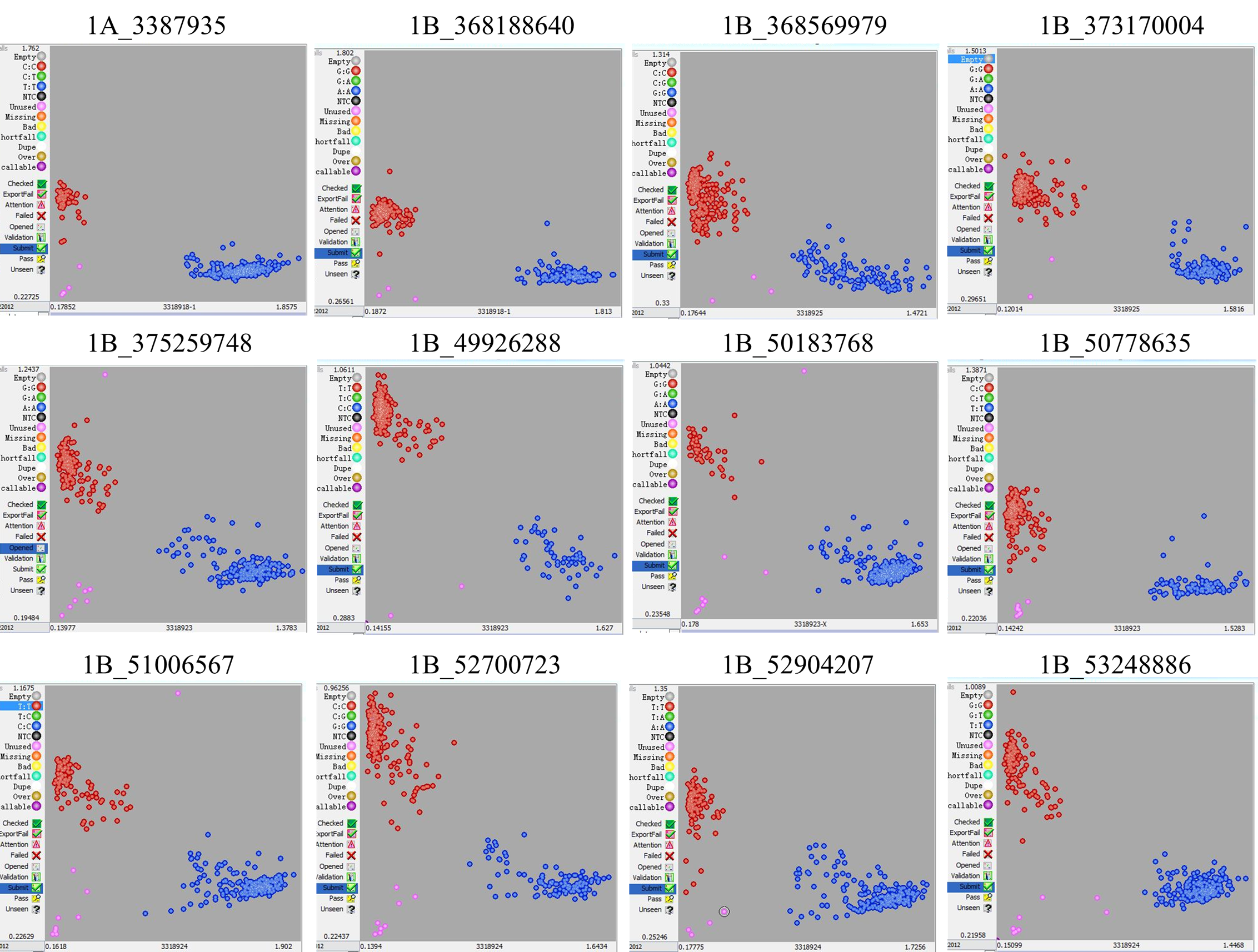

Supplement: Supplementary file 4 — Fig. S3 Genotyping results of 12 KASP markers developed for QTL validation by wheat natural populations. (TIFF 3297 kb) [file 122_2019_3410_MOESM4_ESM.tif]
